# Effect on cell survival and cytoophidium assembly of the adRP-10-related IMPDH1 missense mutation Asp226Asn

**DOI:** 10.3389/fcell.2023.1234592

**Published:** 2023-09-04

**Authors:** Gerson Dierley Keppeke, Chia-Chun Chang, Ziheng Zhang, Ji-Long Liu

**Affiliations:** ^1^ Departamento de Ciencias Biomédicas, Facultad de Medicina, Universidad Católica del Norte, Coquimbo, Chile; ^2^ Rheumatology Division, Escola Paulista de Medicina, Universidade Federal de Sao Paulo, Sao Paulo, Brazil; ^3^ School of Life Science and Technology, ShanghaiTech University, Shanghai, China; ^4^ Institute of Biotechnology, National Taiwan University, Taipei, Taiwan; ^5^ Department of Physiology, Anatomy and Genetics, University of Oxford, Oxford, United Kingdom

**Keywords:** IMPDH1, cytoophidium, apoptosis, cell survival, autosomal dominant retinitis pigmentosa subtype 10 (adRP-10), missense mutation Asp226Asn, rs121912550

## Abstract

**Introduction:** Inosine monophosphate dehydrogenase 1 (IMPDH1) is a critical enzyme in the retina, essential for the correct functioning of photoreceptor cells. Mutations in IMPDH1 have been linked to autosomal dominant retinitis pigmentosa subtype 10 (adRP-10), a genetic eye disorder. Some of these mutations such as the Asp226Asn (D226N) lead to the assembly of large filamentous structures termed cytoophidia. D226N also gives IMPDH1 resistance to feedback inhibition by GDP/GTP. This study aims to emulate the adRP-10 condition with a long-term expression of IMPDH1-D226N *in vitro* and explore cytoophidium assembly and cell survival. We also assessed whether the introduction of an additional mutation (Y12C) to disrupt the cytoophidium has an attenuating effect on the toxicity caused by the D226N mutation.

**Results:** Expression of IMPDH1-D226N in HEp-2 cells resulted in cytoophidium assembly in ∼70% of the cells, but the presence of the Y12C mutation disrupted the filaments. Long-term cell survival was significantly affected by the presence of the D226N mutation, with a decrease of ∼40% in the cells expressing IMPDH1-D226N when compared to IMPDH1-WT; however, survival was significantly recovered in IMPDH1-Y12C/D226N, with only a ∼10% decrease when compared to IMPDH1-WT. On the other hand, the IMPDH1 expression level in the D226N-positive cells was <30% of that of the IMPDH1-WT-positive cells and only slightly higher in the Y12C/D226N, suggesting that although cell survival in Y12C/D226N was recovered, higher expression levels of the mutated IMPDH1 were not tolerated by the cells in the long term.

**Conclusion:** The IMPDH1-D226N effect on photoreceptor cell survival may be the result of a sum of problems: nucleotide unbalance plus a toxic long-life cytoophidium, supported by the observation that by introducing Y12C in IMPDH1 the cytoophidium was disrupted and cell survival significantly recovered, but not the sensibility to GDP/GTP regulation since higher expression levels of IMPDH1-D226N were not tolerated.

## 1 Introduction

Inosine monophosphate dehydrogenase 1 (IMPDH1) is a crucial enzyme in the retina that plays a critical role in the survival and function of photoreceptor cells, as the demand of cGMP for the phototransduction process is very high. These cells are responsible for converting light into electrical signals, and any dysfunction or loss of these cells can lead to severe vision impairments. In photoreceptor cells, the isoform 1 of IMPDH is predominant (Bowne et al., 2006a; Keppeke et al., 2021), probably the reason why mutations in this protein have little effect in other tissues but strongly affect long-term survival in photoreceptors (Aherne et al., 2004). A recent review on the topic can be found elsewhere (Burrell and Kollman, 2022).

Retinal photoreceptor cells contain cytoophidia, which are long, filamentous structures made up in retinal tissue of IMPDH1. Cytoophidium assembly regulates the enzyme’s activity (Keppeke et al., 2018; Fernandez-Justel et al., 2019; Plana-Bonamaiso et al., 2020; Burrell et al., 2022) and may increase protein half-life by preventing proteasomal degradation (Chang et al., 2022). Other tissues, cells, and organisms have other enzymes beyond IMPDH that also assemble into the cytoophidium, such as CTPS, P5CS, and PRPS, also for enzyme activity regulation (Hu et al., 2022; Thangadurai et al., 2022; Zhong et al., 2022). In photoreceptor cells, it has been proposed that regulation of IMPDH1 by GDP/GTP allosteric modulation is controlled by phosphorylation *in vivo* to adjust GTP synthesis to different lighting conditions (Plana-Bonamaiso et al., 2020). Cytoophidium assembly makes IMPDH less sensitive to GTP, allowing it to tolerate higher levels of nucleotides, a way to boost *de novo* biosynthesis of GTP (Keppeke et al., 2018; Fernandez-Justel et al., 2019; Burrell et al., 2022).

Mutations in IMPDH1 have been linked to various retinal diseases, including the autosomal dominant retinitis pigmentosa (adRP), a genetic eye disorder that affects approximately 1 in 4000 people (Sullivan et al., 2013). The adRP-10 subtype of this disease is related to mutations in IMPDH1, particularly the D226N mutation (rs121912550), which is the most frequent in this subtype (Schatz et al., 2005; Wada et al., 2005; Ali et al., 2015), although there are several others (Bowne et al., 2002; Kennan et al., 2002). The D226N mutation in IMPDH1 leads the enzyme to pile up and form microfibers or polymers (Labesse et al., 2013; Labesse et al., 2015), which, depending on intracellular molecular crowding and other factors (Chang et al., 2022), will bundle into large macro-filaments, the cytoophidia, which in these conditions (IMPDH1-D226N-induced) cannot be disrupted by guanosine supplementation, i.e., give IMPDH1 resistance to GDP/GTP feedback inhibition (Thomas et al., 2012; Keppeke et al., 2018; Burrell et al., 2022). The current hypothesis is that mutations in IMPDH1 disrupt feedback inhibition (Fernandez-Justel et al., 2019; Burrell et al., 2022) and could lead to the pathology by increasing the enzyme’s activity beyond normal levels or by forming irreversible filaments (Labesse et al., 2013; Chang et al., 2022). Excessive IMPDH1 activity could cause an imbalance in ATP/GTP levels in darkness or result in abnormal cGMP synthesis, a well-known cause of photoreceptor cell damage (Wang et al., 2017). The impaired capacity of adRP10 patients to sense GDP/GTP levels could cause the formation of irreversible protein aggregates as a result of the formation of filaments in sporadic bright light exposure, as recently demonstrated elsewhere (Plana-Bonamaiso et al., 2020).

Previous studies show that the adRP-10-related mutation in IMPDH1, Asp226Asn (D226N), results in “permanent” cytoophidium assembly that cannot be disrupted by guanosine supplementation (Keppeke et al., 2018), meaning the protein is resistant to GDP/GTP inhibition (Burrell et al., 2022). In addition to an unbalanced nucleotide pool, this could result in the gradual cytoplasmic accumulation of protein aggregates that would eventually turn toxic. Thus, in this study, we will emulate the *in vivo* adRP-10 patient condition with a long-term expression of IMPDH1-D226N *in vitro* and explore cytoophidium assembly and cell survival. We will also evaluate if by disrupting the cytoophidium with another mutation in residue Tyr12 (Johnson and Kollman, 2020; Chang et al., 2022), or by trying to rebalance the nucleotide pools with GMPR (Patton et al., 2011; Keppeke et al., 2018), toxicity caused by the D226N mutation would be less severe. Our findings may shed new light on the molecular mechanisms underlying IMPDH1-related adRP-10 D226N mutation and provide insights into potential therapeutic strategies for this disease.

## 2 Materials and methods

### 2.1 Plasmid cloning

The IMPDH1 coding sequence for the canonical 514aa *ß* isoform (NCBI Reference Sequence: NM_000883.4) was PCR-amplified from a human cell line cDNA (HEp-2), with the following primers: F: atg​gcg​gac​tac​ctg​atc​agc; R: gta​cag​ccg​ctt​ttc​gta​aga​gtg, and the GMPR (NCBI Reference Sequence: NM_006877.4), with the following primers: F: atg​ccc​cgc​ata​gat​gcg​gac​c; R: tta​gct​gaa​cac​ggt​gtt​gtg. The genes were inserted into the linearized pCMV3 vector (Sino Biological, China) that contains piggyBac inverted repeats for the transposon system, by using ClonExpress UItra One Step Cloning Kit (C115, Vazyme, China) according to the manufacturer’s protocol. To the IMPDH1 gene, a myc tag was fused at the C′ terminus and a dark red fluorescent molecule (iRFP670, Ex: 643 nm, Em: 670 nm) at the N′ terminus, interleaved by a P2A sequence. To the GMPR gene, a blue fluorescent protein BFP (mTagBFP2, Ex: 399, Em: 454) was added at the N′ terminus, interleaved by a P2A sequence. The final IMPDH1 construct was pCMV_iRFP670_P2A_IMPDH1-Myc. The final GMPR construct was pCMV_BFP_P2A_GMPR.

Site-directed mutagenesis was carried out by linearizing target constructs with primers containing individual point mutations, followed by recirculation of the plasmid with the Gibson assembly system and NEBuilder HiFi Master Mix Kit (E2621, NEB, United States). Gene size, sequence, and point mutations were confirmed by agarose gel and Sanger sequencing.

Alternatively, for generation of the 546aa splicing isoform α, the 5aa residues at the C′ terminus of the canonical IMPDH1 *ß* were replaced by the 38aa fragment (Plana-Bonamaiso et al., 2020) using NEBuilder HiFi Master Mix. For this experiment, a plasmid containing GFP as a reporter gene was used. The final construct was pCMV_GFP_P2A_Flag-IMPDH1 (β/α).

### 2.2 Cell culture (transfection and selection)

HEp-2 cells (CCL-23, ATCC) were grown to confluence with the culture medium DMEM supplemented with 2 mM L-glutamine and 1% antibiotic–antimycotic (Thermo Fisher Scientific), plus 10% fetal bovine serum (Cultilab, Brazil). The cells were cultured in a 37°C humid incubator with 5% CO_2_.

All transfections were carried out with TurboFect or Lipofectamine 3000 transfection reagents (R0531 or L3000001, Thermo Fisher Scientific) diluted in Opti-MEM I Reduced Serum Medium (31985-070, Gibco, USA), according to instructions provided by the manufacturer.

For transient overexpression, plasmids were transfected alone or mixed (as indicated in the Results) and the cells analyzed 24–48 h after transfection. For generation of cell lines with stable overexpression of the proteins, plasmids were transfected together with a transposase vector (System Biosciences, USA), and after 48 h, the cells were selected with 100 μg/mL of hygromycin B for 2–3 weeks (Figure 3A) before analysis.

For preparation of the IMPDH inhibitor drugs, ribavirin 250 mg pills (Farmanguinhos Laboratory, Brazil) were diluted in water and filtered (0.22 µm) for a 100 mM stock solution. For MPA, mycophenolate sodium 360 mg pills (Myfortic, Novartis Pharma, Switzerland) were diluted in PBS and filtered to obtain a 100 mM stock solution. Drugs were added to the cells 4 h before fixation in a 1/1000 final dilution, or otherwise as indicated in the Results.

### 2.3 Indirect immunofluorescence

For analysis of cytoophidium, the cells grown in 13-mm round coverslips were fixed with 4% paraformaldehyde and probed with antibodies in an indirect immunofluorescence assay, as previously described (Keppeke et al., 2018; Keppeke et al., 2022).

Primary antibodies used: rabbit polyclonal anti-IMPDH2 antibody (12948-1-AP, ProteinTech), mouse anti-Myc monoclonal antibody 9E10 (sc-40, Santa Cruz Biotech), and mouse anti-Flag monoclonal antibody clone M2 (F1804, Sigma). Secondary antibodies used: Cy3-conjugated donkey anti-mouse IgG (#715-165-151, Jackson ImmunoResearch) and Alexa Fluor 488-conjugated donkey anti-rabbit IgG (#A-21206, Invitrogen). After immunofluorescence labeling, the cells were covered with VECTASHIELD containing DAPI (Vector Labs, USA), and the images were captured either by a fluorescence microscope with 200 × or 400 × magnification (Axio Imager. M2, Carl Zeiss, Germany) or by a confocal microscope (LSM 800, Carl Zeiss, Germany).

### 2.4 Cytometry analysis

Cytometry analysis of the reporter genes (iRFP670 and BFP) was performed as previously described (Keppeke et al., 2023). Briefly, the cells were resuspended with trypsin, washed once with PBS, and immediately analyzed in a CytoFLEX cytometer (Beckman Coulter, USA), without fixation. One hundred thousand valid events gated from FSC-SSC were collected for each sample.

### 2.5 Apoptosis analysis

Two methods were applied for analyzing apoptosis. First, the cells were labeled with Annexin V conjugated to Alexa Fluor 488 and propidium iodide (#V13241, Thermo Fisher Scientific), following the manufacturer`s protocol.

Apoptosis was also measured by transfecting the cells 24 h before analysis with a plasmid containing a caspase-3 cleavage sequence, named FlipGFP, as detailed elsewhere (Zhang et al., 2019). After cleavage by activated caspase-3, the reporter GFP became fluorescent. The plasmid was a gift from Xiaokun Shu (Addgene plasmid #124428) (Zhang et al., 2019); however, to apply in our experiments, we replaced the original fluorescent molecule for mTagBFP, and the final plasmid configuration was pSFFV-FlipGFP (Casp3 cleavage seq)_T2A_mTagBFP.

As a positive control, the cells were treated with 0.5 µM of staurosporine for 4 h before analysis. In addition, another positive control was induction of apoptosis by exposing the bare cells to UV light for 1 min, followed by analysis 24 h later.

### 2.6 Data analysis

Images captured using the microscope were analyzed using ImageJ 1.53 software. The proportion of cells presenting a given characteristic, such as cytoophidium, was obtained by quantification of at least two randomly captured images (>100 cells) in each of at least two independent experiments.

All cytometry data were analyzed with CytExpert v2.3 software. Plasmid construction was designed, and DNA sequencing results were analyzed with SnapGene v3.2.1 software.

The data are presented as mean plus error bars indicating standard deviation (S.D.) or standard error of the mean (S.E.M.) as described in the figure legends. For statistical comparisons, if the data did not require sample pairing, an unpaired two-tailed *t*-test with Welch`s correction was applied. If the data require sample pairing, first the repeated measures one-way ANOVA (RM-ANOVA) was applied to assess if the difference among the groups was significant. If it was, comparisons among two groups were made by a two-tailed paired *t*-test (normal distribution) or Wilcoxon matched-pairs signed rank test (not normal). Normal distribution was evaluated by the D'Agostino and Pearson normality test.

Comparisons among each condition and the reference (IMPDH1-WT) are shown above each data-bar, as indicated by the red arrows in the graphs (Figures 4K, L; Figure 5D). Other comparisons among the groups are indicated in the graphs by the horizontal dash. When normalization to the reference (IMPDH1-WT) was applied in each experimental repeat (n = 14) (Figures 2F–H; Figures 4K, L; Figure 6), all statistical analyses were performed with the raw data, without any normalization. *p* ≤ 0.05 was considered statistically significant. All statistical analyses were performed with GraphPad Prism v7.0 software.

## 3 Results

### 3.1 Point mutation D226N in IMPDH1 promotes cytoophidium assembly

**FIGURE 1:**
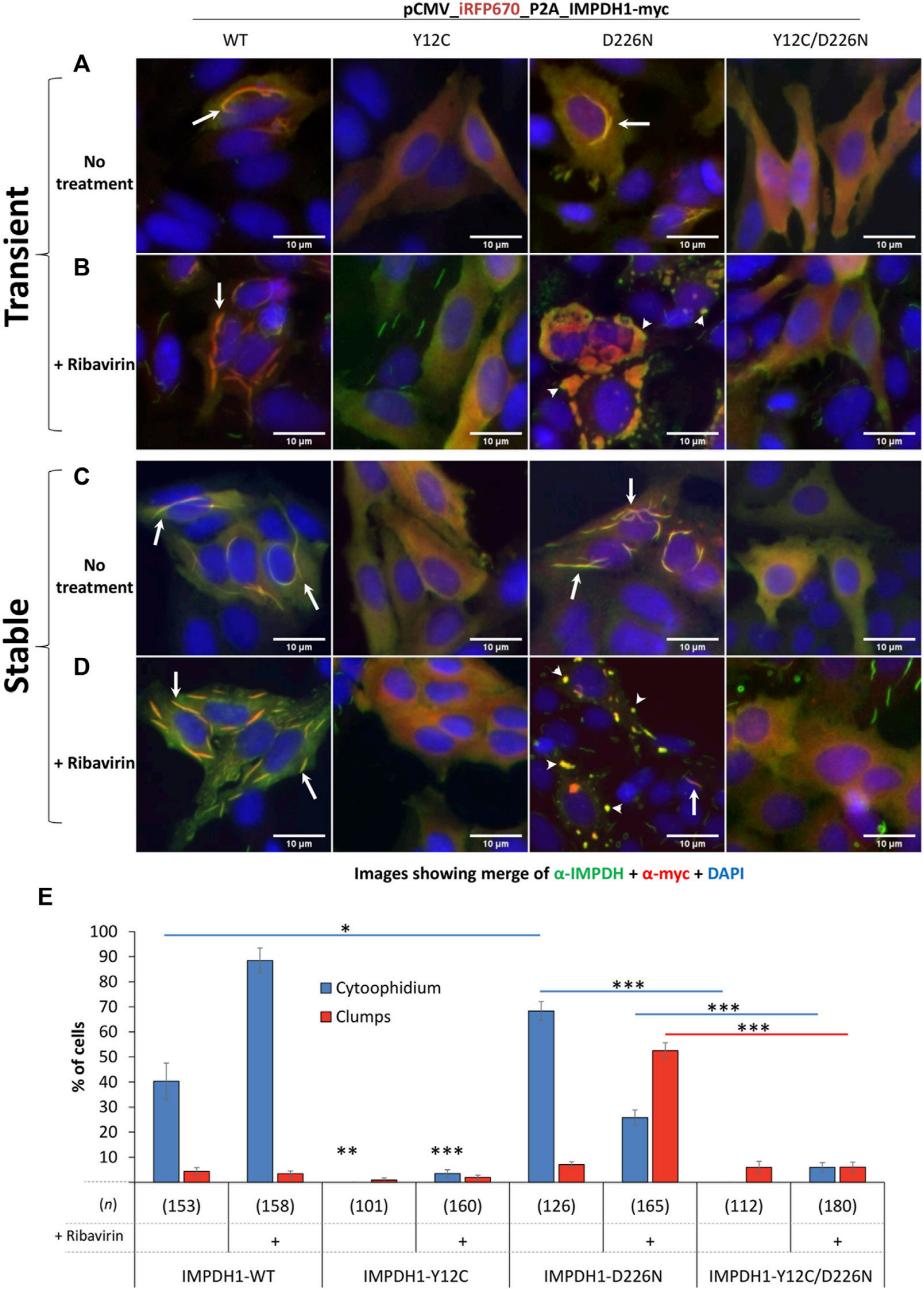
Overexpression of IMPDH1 with point mutations in HEp-2 cells. Transient: **(A, B)** the human IMPDH1 construct was overexpressed in HEp-2 cells for 24 h before IIF. Stable: **(C, D)** after transfection, cells were selected with hygromycin B for 2 weeks before analysis. **(B, D)** Ribavirin (100 µM) was added 4 h before cell fixation. The panels show the merged channels of anti-IMPDH antibody labeling (green) plus the anti-myc labeling (red) plus DAPI (blue), and the separated channels are shown in the Supplementary Material. Arrows indicate cytoophidium and arrowheads clumps. Scale bars = 10 µm. **(E)** Quantification of cytoophidium and clumps in cells with transient expression of IMPDH1 with point mutations. Only transfected cells were counted, and the number of cells counted in each group is shown as (*n*). Error bars = S.E.M. *p < 0.05; **p < 0.01; ***p < 0.001.

To better understand the cytoophidium assembly behavior of IMPDH1-D226N, as well as the potential of replacing Tyr12 for another residue (Burrell et al., 2022; Chang et al., 2022), in this case Cys (Y12C), to disrupt the filaments, the plasmids were initially overexpressed in HEp-2 cells in a transient manner. As previously reported in other cell types (Keppeke et al., 2018), transient expression of IMPDH1-WT in HEp-2 cells results in a considerable proportion of cytoophidium-presenting cells, ∼40%, but it increases to ∼90% upon treatment with the IMPDH inhibitor ribavirin. The presence of the Y12C mutation completely disrupts the cytoophidium and prevents ribavirin- or MPA-induced cytoophidium assembly (arrows in Figure 1 and Supplementary Figure S1).

Overexpression of IMPDH1-D226N mutation induces cytoophidium in ∼70% of transfected cells, significantly higher than that of IMPDH1-wt overexpression. Curiously, under ribavirin or MPA treatment, the cytoophidium turns into large clumps in ∼50% of the cells (arrowheads in Figure 1B). The presence of the Y12C mutation disrupts both cytoophidium and clumps induced by the presence of D226N mutation or the drugs (Figures 1A, B, E and Supplementary Figure S1). However, although we name these structures as “clumps,” we cannot exclude the possibility of them being composed of cytoophidium-like microfibers, especially because the presence of the Y12C mutation disrupts the D226N-induced clumps just like it does for the cytoophidium (Figure 1B).

### 3.2 Building the model for stable expression of mutated IMPDH1

**FIGURE 2:**
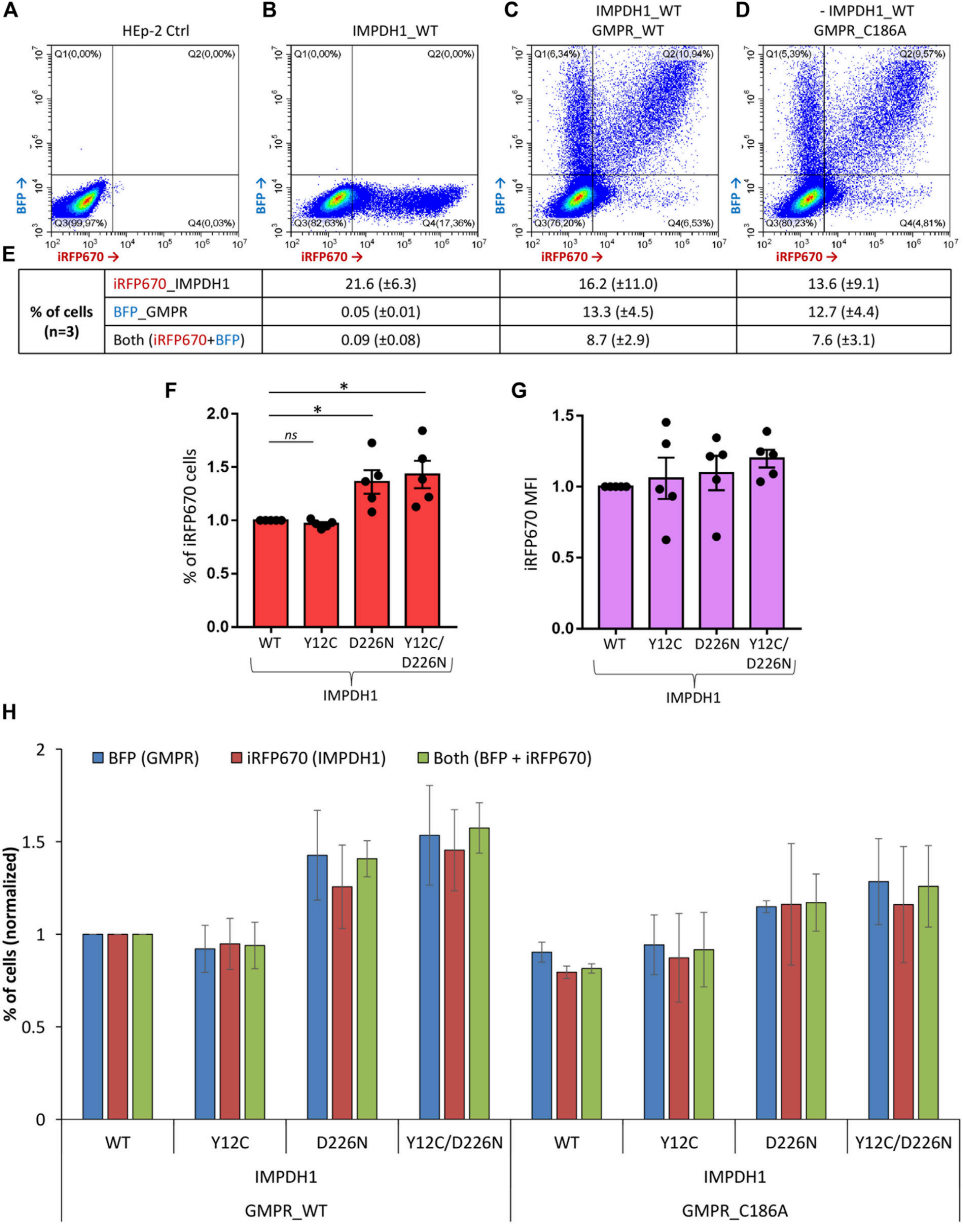
Transient transfection efficiency for all plasmids. Cells were transfected with iRFP670_P2A_IMPDH1 alone or in addition to BFP_P2A_GMPR and analyzed 24 h later. **(A–D)** Representative panels of the cytometry analysis of iRFP670 and BFP fluorescence. ∼80 k events shown in each panel. **(E)** Averages of cells expressing the given plasmids in **(B–D). (F, G)** Cells were transfected with iRFP670_P2A_IMPDH1 plasmid alone. Proportion of cells expressing iRFP670 **(F)** or the mean fluorescence intensity (MFI) of iRFP670 **(G)** was normalized to IMPDH1-WT in each experiment repeat (n = 5). **(F)** *p < 0.05. **(G)** RM-ANOVA *p* = 0.366. **(H)** Proportions of cells expressing BFP, iRFP670, or both were normalized in each experiment repeat (n = 3) to IMPDH1_WT + GMPR_WT. RM-ANOVA analysis showed no difference among the groups regarding proportion of cells expressing iRFP670 (*p* = 0.352), or BFP (*p* = 0.070), or both (*p* = 0.137). Error bars = S.E.M.

In our experiments, transient transfection efficiency in the HEp-2 cells ranged from ∼10% when two plasmids were transfected together, up to 30% when it was only one plasmid (Figures 2A–G). In addition, the proportion (%) of cells expressing the dark-red fluorescent reporter gene iRFP670 (see methods), referred here as iRFP670-positive cells, as well as the reporter mean fluorescence intensity (MFI), showed similarities or higher values when expressing IMPDH1-D226N in comparison with IMPDH1-wt (Figures 2F, G), suggesting that in the short 1–2 day period of transient overexpression, D226N did not cause considerable damage to the cells.

**FIGURE 3:**
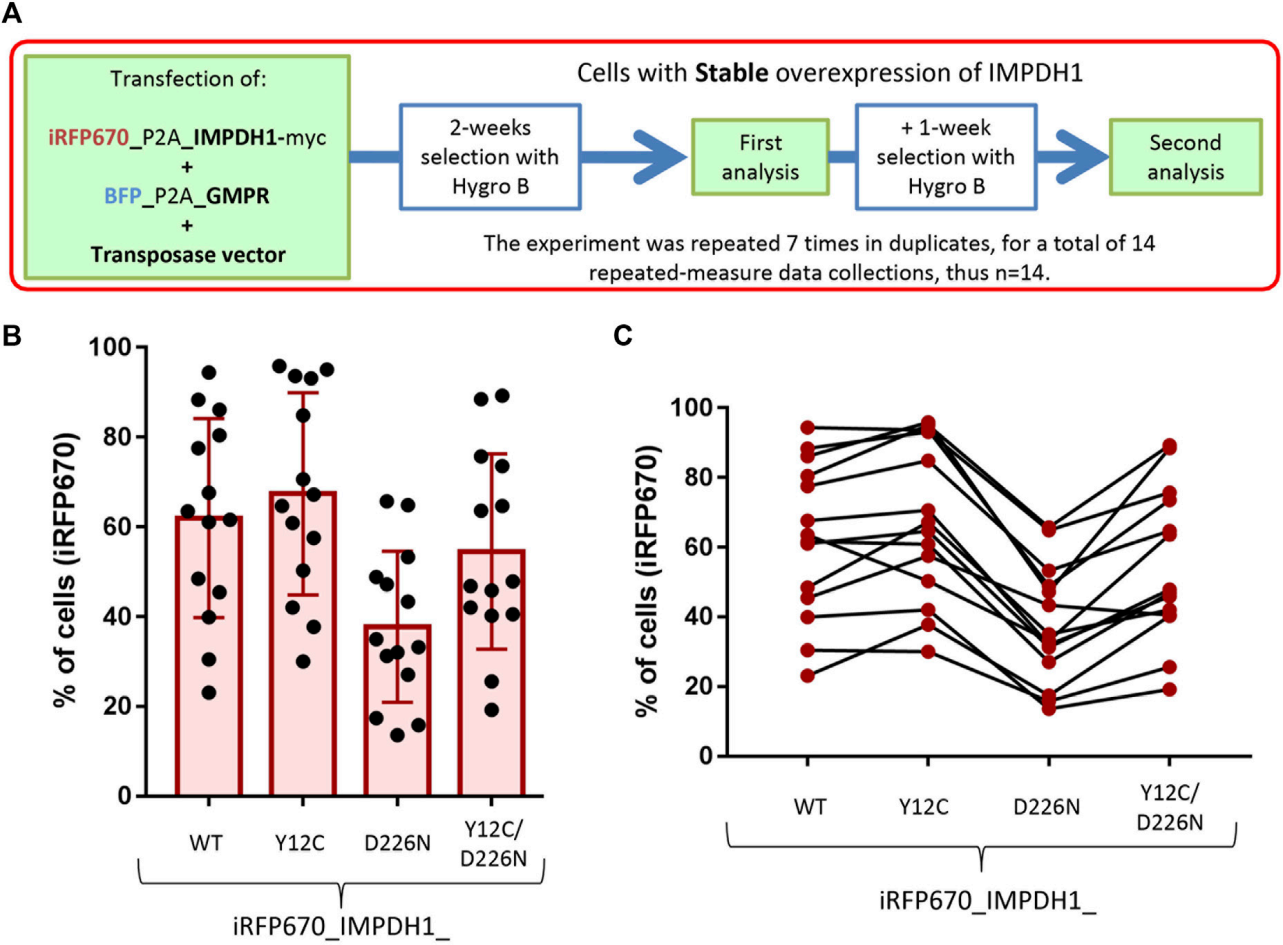
Generation of cell lines with stable expression of IMPDH1 and GMPR. **(A)** Strategy for generation and analysis of the cell lines with stable expression of iRFP670_P2A_IMPDH1 alone or in addition to BFP_P2A_GMPR. **(B, C)** Proportion of cells overexpressing iRFP670_IMPDH1 after selection. **(B)** Distribution of proportions where each dot represents one measure. **(C)** Consistency, where each line represents one data collection. Error bars = S.D.

To evaluate how the adRP-10-related IMPDH1-D226N mutation affects cell survival in a longer period, we selected the cells with hygromycin B for 2–3 weeks to generate stable cell lines expressing the mutant IMPDH1 (Figure 3A). Regarding the cytoophidium assembly, stable expression of IMPDH1_WT, Y12C, D226N, or Y12C/D226N showed a similar behavior as the transient expression (Figures 1C, D and Supplementary Figure S2), meaning the D226N-induced cytoophidium clumped in the presence of ribavirin, and Y12C prevented all cytoophidium assembly.

### 3.3 D226N mutation in IMPDH1 affects long-term cell survival

By analyzing the percent of iRFP670, we could estimate if the presence of D226N mutation affected cell survival. If overexpression of IMPDH1-D226N represents a competitive disadvantage to the cells, after the selection period, the proportion of cells expressing the mutated IMPDH1 would be lower than that of IMPDH1-WT. In all cell lines, proportions of iRFP670-positive cells varied considerably, from ∼20% up to ∼100% (Figure 3B); however, there was a trend among each experimental batch, meaning if the percent of positive cells was higher for one plasmid, it would also be higher for the others (Figure 3C).

**FIGURE 4:**
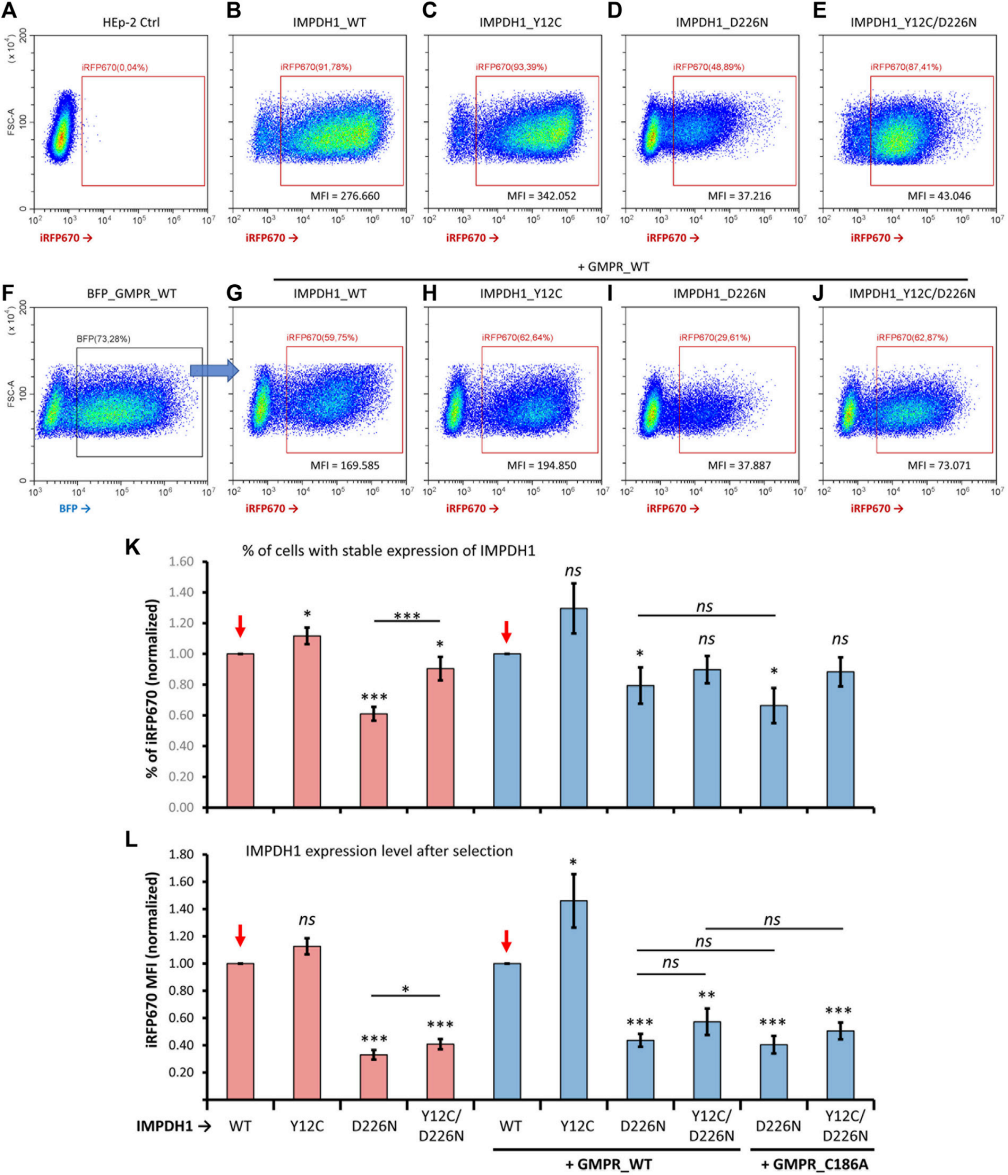
Comparisons of proportion of cells overexpressing mutated IMPDH1 after selection. **(A–E)** Representative cytometry panels of the proportion of cells expressing mutated IMPDH1 after selection with hygromycin **(B)**. Cells transfected with the iRFP670_P2A_IMPDH1 plasmid alone. **(F)** Cells with simultaneous transfection of IMPDH1 and GMPR were gated for BFP before analysis of iRFP670 **(G–J)** Overexpression of mutated IMPDH1 in cells also expressing GMPR-WT. **(A–J)** The ∼30–50k events shown in each panel. **(K)** Average proportion of iRFP670_IMPDH1-expressing cells among experimental repeats, and **(L)** average mean fluorescence intensity (MFI) of iRFP670. Values in each data collection (n = 14) were divided (normalized) by those of IMPDH1-WT, as indicated by the red arrows. Error bars = S.E.M. *p < 0.05; **p < 0.01; ***p < 0.001; *ns* = not significant.

**FIGURE 5:**
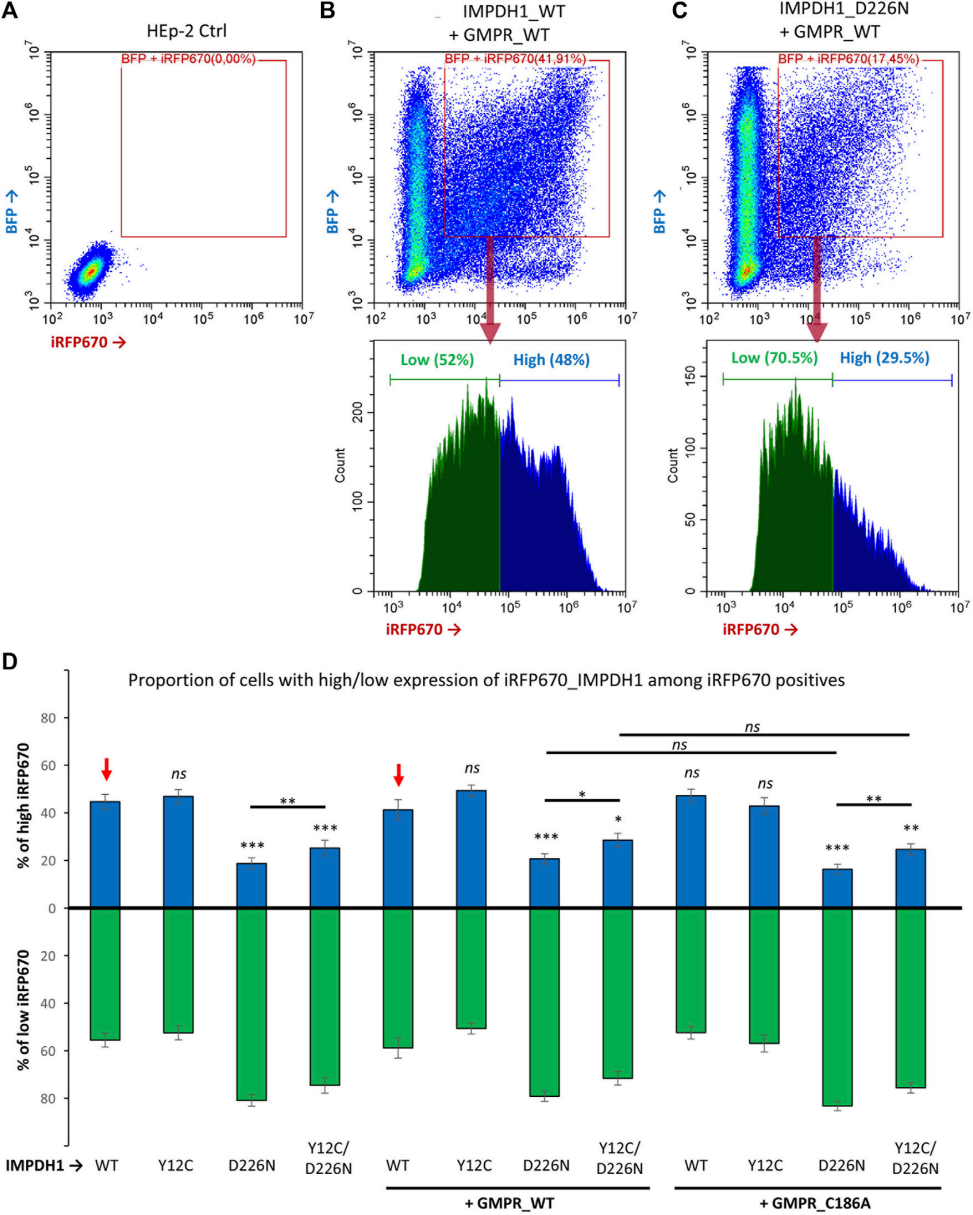
Proportion of cells expressing high levels of IMPDH1. Among iRFP670 positives, alone or including BFP positives, the proportion of cells expressing high or low levels of iRFP670 was evaluated. **(A–C)** Representative cytometry panels with the analysis strategy. The “cut-line” for high iRFP670 expression was defined for IMPDH1_WT as ∼45–50% in each experimental repeat (n = 14) and applied for the other conditions. **(D)** Average proportion of cells expressing high (blue bars) or low (green bars) levels of iRFP670. For statistical analysis, values in each condition were compared to those of IMPDH1-WT, as indicated by the red arrows. Error bars = S.E.M. *p < 0.05; **p < 0.01; ***p < 0.001; *ns* = not significant.

For better comprehension of the effect of D226N in cell survival, the percent of positive cells was normalized in each experimental repeat (n = 14), as shown in Figure 4K. The average percent of iRFP670-positive cells in IMPDH1-WT was 62%, but in IMPDH1-D226N, it was 37.8% (Table 1), a decrease of ∼40% in the percent of iRFP670-positive cells (Figures 4A–D, K). In addition, we also evaluated the percent of cells with high overexpression of iRFP670_IMPDH1 among the iRFP670-positive cells, and the percent of high iRFP670 in IMPDH1-D226N was significantly lower (18.7%) when compared to IMPDH1-WT (44.7%) (Table 1; Figure 5).

**TABLE 1:** Stable overexpression of mutated IMPDH1 (n = 14).

Plasmid			Percent of iRFP670			Percent of high iRFP670 among iRFP670 positives			iRFP670 mean fluorescence intensity			Percent of BFP		
BFP_GMPR	iRFP670_IMPDH1			^a^	^a^		^a^	^a^		^a^	^a^		^a^	^a^
-	WT		62.0 ± 5.9	***	**	44,7 ± 3.1	***	***	162.533 ± 16.614	***	***	-		
	Y12C		67.4 ± 6.0			46,9 ± 2.9			174.794 ± 14.441			-		
	D226N		37.8 ± 4.5			18,7 ± 2.4			47.944 ± 3.945			-		
	Y12C/D226N		54.5 ± 5.8			25,3 ± 3.3			62.923 ± 6.944			-		
GMPR_WT	WT	^b^	55.0 ± 5.9	*		41,3 ± 4.3	***		158.754 ± 17.559	***		53.6 ± 5.7	*ns*	*ns*
	Y12C		61.9 ± 4.8			49,4 ± 2.3			201.646 ± 17.474			54.7 ± 5.0		
	D226N		42.5 ± 6.8			20,7 ± 2.1			60.685 ± 4.646			57.5 ± 5.2		
	Y12C/D226N		50.3 ± 7.4			28,6 ± 2.9			84.215 ± 13.239			59.6 ± 5.3		
GMPR_C186A	WT		69.0 ± 5.1	**		47,2 ± 2.7	***		221.655 ± 24.446	***		50.5 ± 7.0	*ns*	
	Y12C		62.5 ± 5.0			42,9 ± 6.5			188.555 ± 29.453			59.9 ± 5.5		
	D226N		37.5 ± 7.7			16,3 ± 2.1			54.753 ± 6.915			60.2 ± 6.2		
	Y12C/D226N		45.7 ± 5.5			24,7 ± 2.3			71.224 ± 7.409			60.7 ± 5.3		

^a^ Repeated measures one-way ANOVA (RM-ANOVA) analysis. *p < 0.05; **p < 0.01; ***p < 0.001; *ns* = not significant.

^b^ Cells selected for BFP positivity before analysis of iRFP670.

Since the reporter gene was fused to the IMPDH1 interleaved by a P2A sequence, by analyzing the iRFP670 mean fluorescence intensity (MFI) in the positive cells, we could estimate the expression level of IMPDH1, estimating the amount of protein that was not toxic enough to prevent long-term cell survival. The average MFI in the D226N-positive cells was <30% of that of the IMPDH1-WT-positive cells (Table 1; Figure 4L). Altogether, these data suggest that the presence of increased amounts of IMPDH1 with the D226N mutation significantly affects long-term cell survival.

### 3.4 Combined effect of D226N and Y12C in IMPDH1 on cell survival

We then evaluated if the presence of Y12C mutation that disrupts cytoophidium (Figure 1C) could decrease the D226N toxicity and improve long-term cell survival. The percent of cells expressing Y12C/D226N was significantly higher (54.5%) that that of IMPDH1-D226N (37.8%) (Table 1; Figure 3), which represents 0.9 of the IMPDH1-WT (Figures 4B, E, K).

However, when we analyzed the MFI and the percent of high iRFP670, there was only a slight although significant increase in the Y12C/D226N compared with the D226N alone (Table 1; Figure 4L; Figure 5), with values significantly lower than those of the WT. This suggests that although cell survival (% of iRFP670) of Y12C/D226N was recovered, higher expression levels of the mutated IMPDH1 were not tolerated by the cells in the long term.

We hypothesized that the improved cell survival in Y12C/D226N when compared with the D226N alone could be, in part, credited to the presence of the cytoophidium-disrupting Y12C since in all analyses of percent of iRFP670 and MFI, the cells expressing IMPDH1-Y12C tolerate slightly increased expression levels of protein when compared with IMPDH1-WT (Figures 4K, L).

### 3.5 Contribution of GMPR to cell survival

One of the hypotheses for the toxic effect of D226N mutation on IMPDH1 is that it may disrupt feedback inhibition of IMPDH1 by GDP/GTP (Burrell et al., 2022), which is the reason guanosine supplementation cannot disassemble D226N-induced cytoophidium (Keppeke et al., 2018), resulting in a nucleotide imbalance with high levels of the pathway’s product. Thus, in an attempt to rebalance GDP/GTP pools and improve cell survival, we overexpressed GMPR, an enzyme that converts GMP back to IMP. In a previous study from our group (Keppeke et al., 2018), we demonstrated that GMPR overexpression boosts IMPDH activity, resulting in extensive cytoophidium assembly. GMPR is not sensitive to feedback inhibition by its product; thus, overexpression of the enzyme is very likely to result in accumulation of IMP (Patton et al., 2011). The proportion of cells co-expressing GMPR and the IMPDH1 mutant plasmids in a transient 1–2 day time was similar under all conditions (Figure 2H).

In the long-term cell-lines, percent of D226N-expressing cells among the GMPR-WT positive cells was 42.5% (Table 1); this represents 0.77 of IMPDH1-WT + GMPR-WT (Figures 4F–K); however, this was not different from D226N + GMPR-C186A. This mutation on GMPR, C186A, renders the enzyme catalytically dead (Li et al., 2006; Patton et al., 2011). In fact, by combining the Y12C/D226N with GMPR-WT or GMPR-C186A, the percent of iRFP670-positive cells was similar to that of the IMPDH1-WT, without statistical difference, suggesting cell survival was fully recovered under these conditions, overcoming the D226N toxicity (Table 1; Figures 4G–K).

Similar to when expressing mutated IMPDH1 alone, in the presence of GMPR, percent of high iRFP670 presented a slight although significant increase for Y12C/D226N, but still significantly lower than that of IMPDH1-WT + GMPR-WT (Table 1; Figure 5). In addition, the iRFP670 MFI in Y12C/D226N + GMPR-WT was similar to that in D226N + GMPR-WT (Figure 4L). Altogether, although GMPR may contribute together with the Y12C to recover cell survival, higher expression levels of IMPDH1-D226N were still not tolerated by the cells even with GMPR overexpression.

### 3.6 D226N mutation in IMPDH1 promotes apoptosis

**FIGURE 6:**
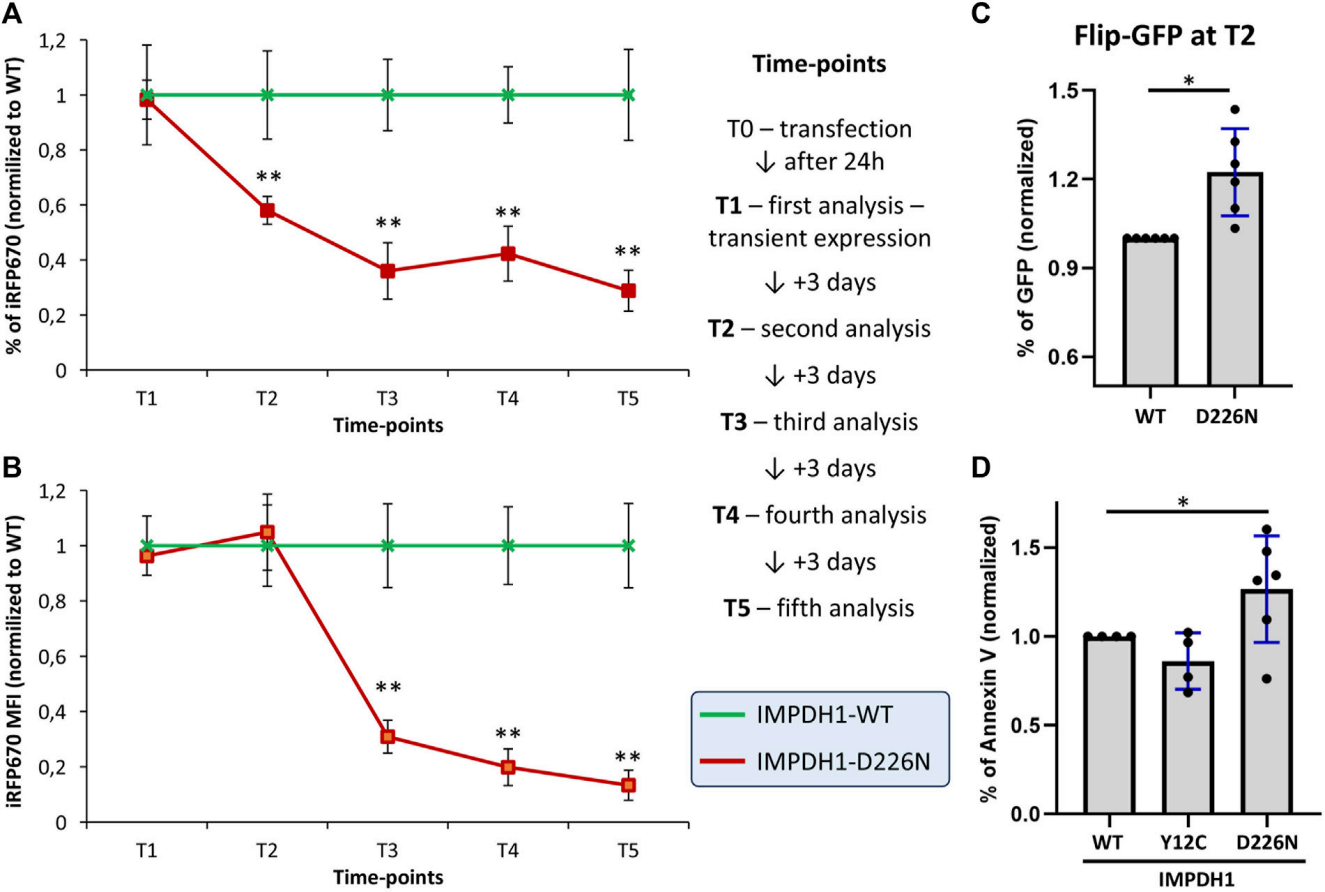
Cell death dynamics in the presence of IMPDH1-D226N. **(A)** Proportion of cells expressing iRFP670 or **(B)** the iRFP670 MFI was analyzed in different time-points after transfection, without hygromycin B treatment. **(C)** Four days after transfection of IMPDH1-WT or D226N, without hygromycin treatment, apoptosis was analyzed with FlipGFP. The plasmid pSFFV-Flip-GFP-T2A-BFP was transfected 24 h before analysis. Cells were first gated for iRFP670 (IMPDH1), followed by BFP, followed by GFP (see Supplementary Figures S3E–I for details). **(D)** Apoptosis was also labeled by Annexin V in the cell-lines with stable expression of IMPDH1 (see Figure 3A and Supplementary Figures S3A–D). In all panels, D226N values were normalized for IMPDH1-WT in each experimental batch (n = 6), and the average is shown. Error bars = S.D. *p < 0.05; **p < 0.01.

Either an accumulation of protein aggregates or an unbalanced pool of nucleotides would result in cell damage and death by apoptosis. To evaluate the dynamics of cell death induced by the presence of D226N without the effect of hygromycin B treatment, the percent of iRFP670-expressing cells was analyzed 24 h after transfection (T1) and within 3-day intervals (time-points) up to 2 weeks (Figure 6). Within ∼1 week (time-point T3), the percent of iRFP670-positive cells was <50% of the IMPDH1-WT, and this was maintained in the following time-points (Figure 6A). Curiously, at time-point T2 (4 days after transfection), iRFP670 MFI was similar in D226N and WT, but the percent of positive cells decreased to ∼40%, and only at time-point T3 (7 days after transfection), the MFI decrease ∼70% in D226N when compared with WT (Figures 6A, B).

To measure apoptosis, we used a plasmid containing a caspase-3 cleavage sequence, named FlipGFP, as detailed elsewhere (Zhang et al., 2019). The gating strategy is detailed in Supplementary Figures S3E–I. With this method, the cells were analyzed at time-point T2 (4 days after transfection and without hygromycin). The percent of GFP-positive cells, indicating the apoptosis rate, as well as the GFP MFI, was 22% and 23% higher, respectively, in D226N than in WT (Figure 6C and Supplementary Figure S3J).

In the cells with stable expression of IMPDH1-WT or D226N, apoptosis was labeled for phosphatidylserine translocation with Annexin V (Supplementary Figure S3A–D). In all experiments, control HEp-2 cells without any transfection would present some cells in apoptosis, usually ≤5% of the cells, and the same was observed in cells expressing IMPDH1-WT (Supplementary Figure S3). The percent of cells labeled by Annexin V was 27% higher in D226N than in WT (Figure 6D and Supplementary Figure S3J).

Altogether, the presence of IMPDH1-D226N results in a ∼20–30% increase in the apoptosis rate, with a cumulative effect, meaning within 1 week, the percent of iRFP670-positive cells will reach the ∼40–50% decrease observed in the D226N cell lines when compared with IMPDH1-WT.

### 3.7 IMPDH1 retinal dominant splicing isoform *α*

**FIGURE 7:**
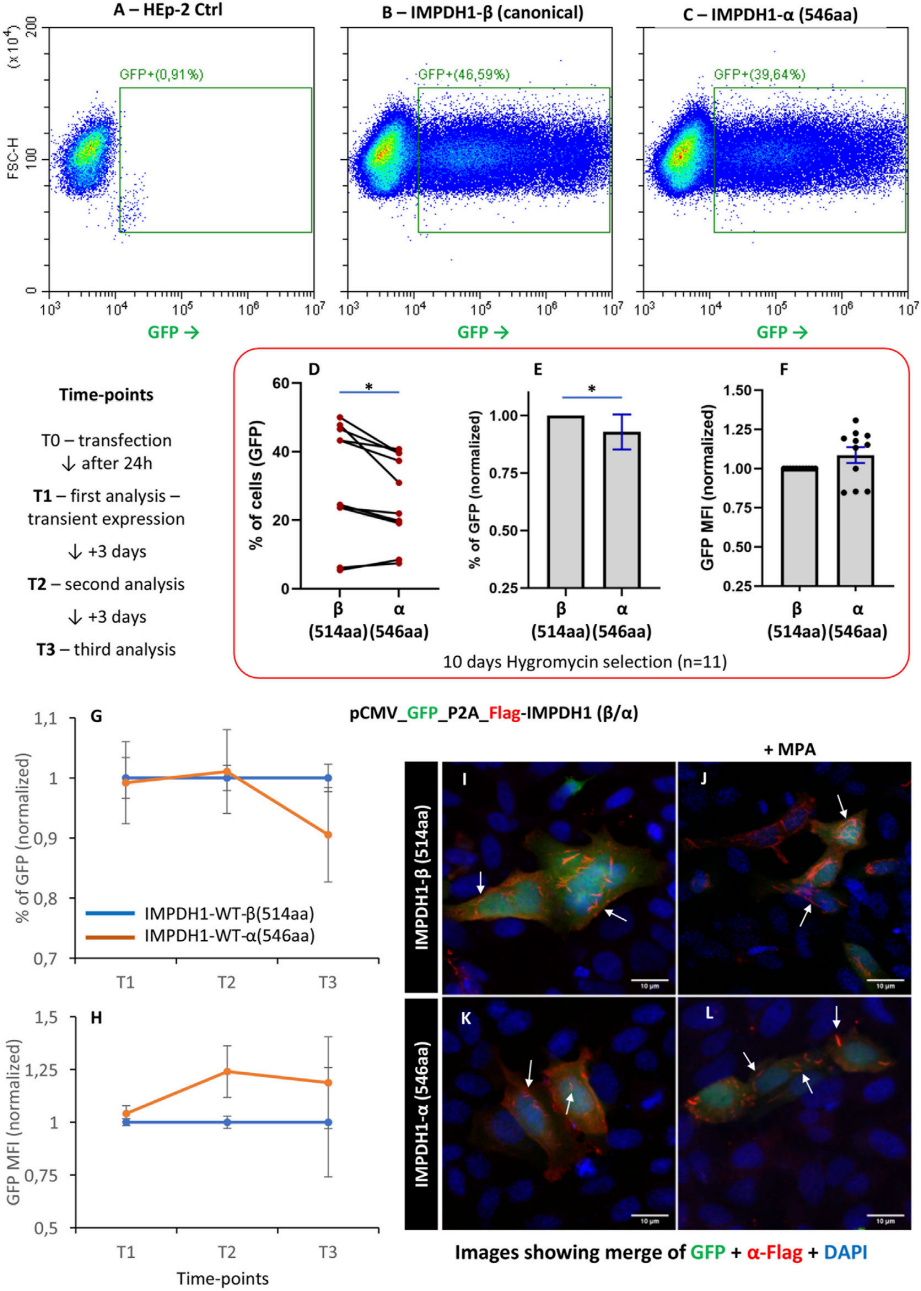
Overexpression of the IMPDH1 retinal dominant splicing isoform α. **(A–F)** Cells were transfected with GFP-P2A-Flag-IMPDH1 (β/α) and selected with hygromycin B for 10 days before analysis (n = 11). **(B, C)** Representative cytometry panels of cells with stable expression of GFP. **(D)** Distribution of percent of GFP-positive cells and consistency, where each line represents one data collection. **(E)** Percent of GFP normalized to IMPDH1-β in each experimental row. **(F)** GFP mean fluorescence intensity, normalized to IMPDH1-β. **(G, H)** Proportion of GFP **(G)** or the GFP MFI **(H)** was analyzed in different time-points after transfection, without hygromycin B treatment (n = 5). **(I–L)** Twenty-four hours after transfection, cells were fixed and labeled with anti-Flag antibody (red). In J and L, cells were treated with MPA (100 µM) for 1 h before cell fixation. Arrows indicate cytoophidia. Scale bar = 10 µm. Error bars = S.E.M. *p < 0.05.

For most of the experiments, we applied the IMPDH1 canonical *ß* isoform, with 514aa. However, in human retinal photoreceptor cells, an alternative splicing isoform *α* is the predominant IMPDH1 isoform expressed (Bowne et al., 2006a). In IMPDH1-α, the 5aa residues at the C′ terminal are replaced by 38aa (Spellicy et al., 2007); thus, this isoform has 546aa, and the additional terminal peptide extensions influence enzyme activity (Andashti et al., 2021). IMPDH-α has a decreased sensitivity to GTP binding when compared to IMPDH1-β (Andashti et al., 2020; Burrell et al., 2022); thus, we question if the long-term HEp-2 cell survival would be affected by the presence of IMPDH1-α(546aa). After transfection of IMPDH1 (β/α), the cells were selected for 10 days with hygromycin for stable expression of the splicing isoform *α* (Figures 7A–F). The proportion of IMPDH1-α(546aa)-expressing cells was ∼0.9 of that of IMPDH1-β(514aa), a significant decrease of ∼10% (Figures 7D, E), although the expression level (estimated by the GFP mean fluorescence intensity) was similar among the isoforms (Figure 7F).

To evaluate the dynamics of cell survival, after transfection, the cells were analyzed at different time-points within 1 week (7 days) without hygromycin selection (Figures 7G, H). A total of 0.9% of cells with expression of isoform *α* in comparison with the canonical *ß* were observed at T3 (day 7) (Figure 7G). In addition, isoform *α* can assemble cytoophidium under MPA treatment, similar to the canonical *ß* (Figures 7I–L).

Altogether, these data show that cell survival was slightly lower in the presence of IMPDH1-α(546aa) when compared with the canonical IMPDH1-β(514aa), a ∼10% decrease, but the intensity of damage is much less severe than that of IMPDH1-D226N. However, since α(546aa) is the major isoform in the human retina, in adRP-10 patients that carry the IMPDH1-D226N mutation, the effect would add up and damage photoreceptor cells even further.

## 4 Discussion

Our data show that the adRP-10-related IMPDH1 missense mutation D226N induces formation of large cytoophidium, and the replacement of tyrosine at position 12 disrupts those cytoophidia, as previously demonstrated elsewhere (Burrell et al., 2022; Chang et al., 2022). The long-term overexpression of IMPDH1-D226N in HEp-2 cells significantly affected cell survival, emulating the *in vivo* side effect of this mutation in retinal photoreceptor cells. We were also able to significantly recover cell survival by introducing another mutation in IMPDH1, the Y12C, which disrupts the filaments.

The impact of the exogenous IMPDH1-Y12C on the polymerization of endogenous IMPDH2 and the assembly of the cytoophidium, which is known as a large bundle of IMPDH polymers, can be attributed to the fundamental understanding that IMPDH polymers are composed of octamers (Burrell and Kollman, 2022). Given the high sequence similarity (84%) between IMPDH1 and IMPDH2, it is plausible that these two isoforms can assemble heterooctamers. Consequently, the Y12C mutant IMPDH1, which disrupts the interface between two neighboring octamers (Anthony et al., 2017; Johnson and Kollman, 2020), exerts a dominant effect on the polymerization of IMPDHs. It is reasonable to suspect that even a small amount of the mutant IMPDH could have significant effects on the cytoophidium, as the presence of the mutant IMPDH at any point of the polymer may weaken interactions and destabilize the long string.

Creating an accurate *in vivo* model that faithfully replicates adRP-10-related IMPDH1 mutations poses significant challenges due to the difficulty of introducing mutations in animals and waiting for the disease phenotype to manifest. *In vitro*, the complex metabolism within photoreceptor cells is also a challenge, as well as retinal cell lines such as the retinal pigment epithelial cell (RPE1) or the 661W cone photoreceptor cell line which grow at a much slower rate than other more traditional tumor-derived cells, such as HeLa and HEK293-T. In our model, we used HEp-2 cells, initially thought to be a lineage of laryngeal carcinoma, but recent evidence suggests that it is derived from a HeLa contamination (Gorphe, 2019). In any case, by overexpressing the mutated IMPDH1 in the long term, we emulate the retinal photoreceptor cells where IMPDH1 is the predominant isoform expressed (Bowne et al., 2006a). However, in photoreceptor cells, an alternative splicing isoform *α* is the predominant IMPDH1 isoform, with 546aa. IMPDH1-α has decreased sensitivity to GTP feedback inhibition when compared with the canonical 514aa IMPDH1-β (Andashti et al., 2020; Burrell et al., 2022). Our results show that long-term cell survival was slightly lower in the presence of IMPDH1-α than in the presence of IMPDH1-β, and models to study adRP-10-related mutations should take this feature into account.

By utilizing this model, we could emulate the long-term expression of IMPDH1-D226N and evaluate cytoophidium assembly, cell tolerance, and survival. However, there are at least 12 mutations in IMPDH1 that are associated to adRP in humans (Bowne et al., 2006b). From those, six have been characterized to provide IMPDH1 resistance to GDP/GTP feedback inhibition (Fernandez-Justel et al., 2019; Johnson and Kollman, 2020; Burrell and Kollman, 2022): N198K, R224P, L227P, D226N, R231P, and K238E. From these findings, we have previously demonstrated that permanent cytoophidia assemble when the mutations R224P, D226N, and R231P are present in IMPDH1 (Keppeke et al., 2018). Future studies should evaluate the potential side effects, such as tolerance and survival, in the long-term expression of the retinal-specific IMPDH1 isoforms containing the other adRP-10-related mutations.

In retinal cells, the presence of cytoophidium is tightly regulated by light conditions, as demonstrated in previous studies (Plana-Bonamaiso et al., 2020; Cleghorn et al., 2022). This regulation is of particular significance in photoreceptor cells where IMPDH1 is highly overexpressed. The isoform 1, predominantly found in photoreceptor cells, exhibits splice variants with C or N-termini extensions (Spellicy et al., 2007), which have been implicated in the reduction of feedback inhibition by GDP/GTP (Andashti et al., 2020; Andashti et al., 2021; Burrell et al., 2022; Burrell and Kollman, 2022). This intricate interplay between cytoophidium assembly, light sensitivity, and IMPDH1 isoforms underscores the complexity of the regulatory mechanisms involved in maintaining nucleotide homeostasis and cellular function in the retina. Understanding these relationships provides valuable insights into the molecular dynamics of photoreceptor cells and may contribute to the development of targeted therapeutic approaches for retinal diseases associated with dysregulated IMPDH1 activity.

In conclusion, we hypothesize that since cytoophidium increases protein half-life by making it difficult to be degraded (Chang et al., 2022), this could be the cause of what we found in this study: increased proportion of cells dying by apoptosis when IMPDH1-D226N was overexpressed in the long term, mimicking the effects of this mutation in retinal photoreceptor cells *in vivo*. The D226N-induced “toxic long-life cytoophidium” hypothesis would be in addition to losing its sensibility to GDP/GTP inhibition (Burrell et al., 2022), which results in nucleotide imbalance, also a cause of cell damage (Wang et al., 2017). Indeed, a combination of issues is more likely, including the imbalance of nucleotides and the presence of the toxic cytoophidium. This presumption is supported by the fact that when Y12C was introduced into IMPDH1-D226N, the disruption of cytoophidium led to a significant recovery in cell survival. Nonetheless, it is plausible that even though the cytoophidium disruption improved cell survival, it did not restore sensitivity to GDP/GTP regulation, which is evident from the fact that increased expression levels of IMPDH1-D226N still remained intolerable. Future research should delve into whether this pattern applies to the other six IMPDH1 mutations characterized by the observed resistance to GTP-feedback inhibition (Burrell et al., 2022), as well as whether our findings hold true *in vivo* using an adRP-10 animal model.

## Data Availability

The raw data supporting the conclusion of this article will be made available by the authors, without undue reservation.
