# Supplementary material for: Effect on cell survival and cytoophidium assembly of the adRP-10-related IMPDH1 missense mutation Asp226Asn
Source: Front Cell Dev Biol. 2023 Sep 4;11:1234592. doi: 10.3389/fcell.2023.1234592 (PMC10507268; doi:10.3389/fcell.2023.1234592)
Supplement: Supplementary file 1 [file DataSheet1.PDF]

SUPPLEMENTARY FILE

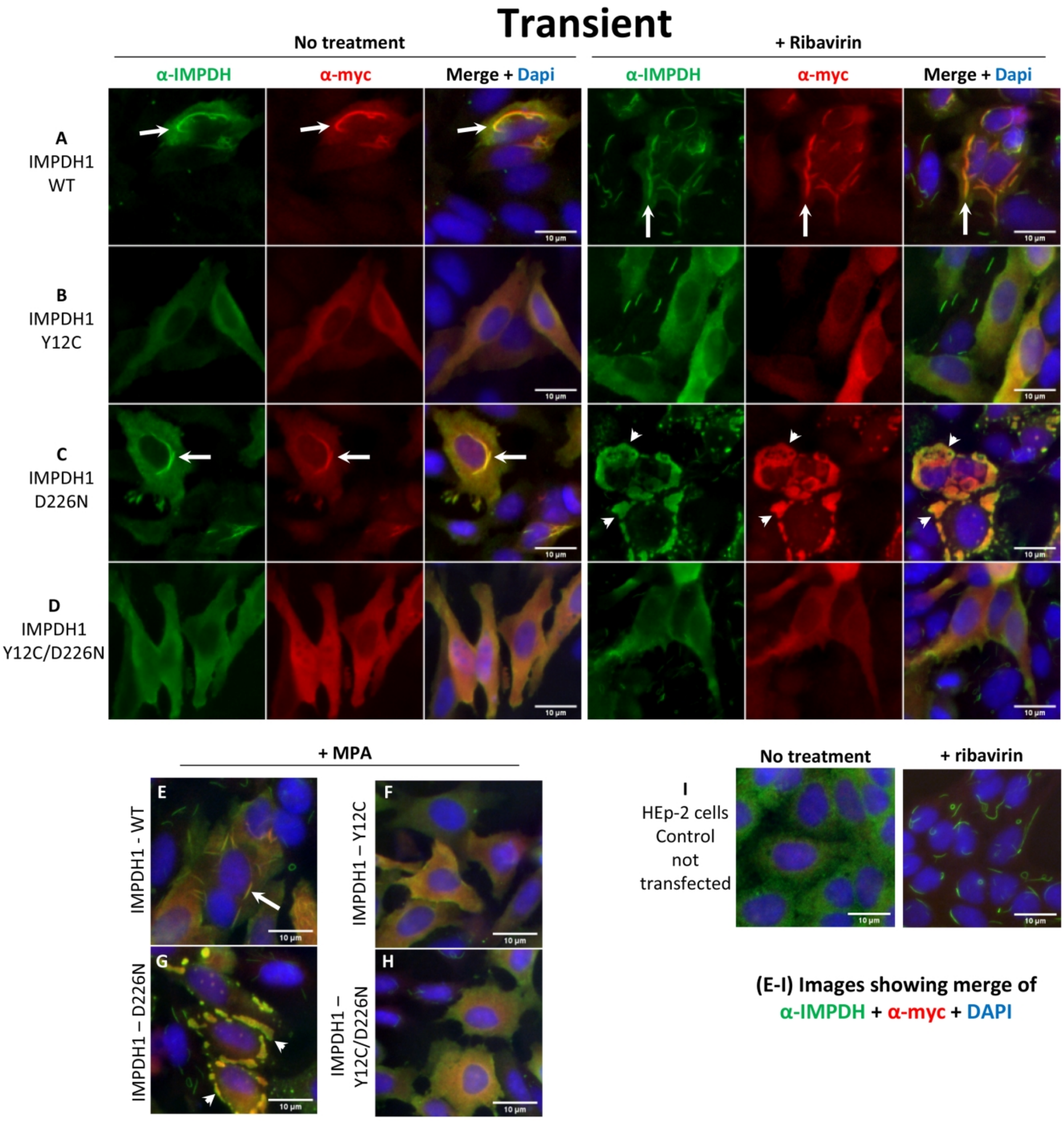

Supp Figure 1. Separate channels for the transient overexpression of IMPDH1 with point mutations. (A-D) The IIF channels of anti-IMPDH antibody labeling (green) plus the anti-myc labeling (red) are shown separately, Merge + DAPI panels are the same as those in Figure 1. (E-H) IMPDH1 construct was overexpressed in HEp-2 cells and treated with MPA (100µM) for 4h before cells fixation. (I) Non-transfected control HEp-2 cells without or with ribavirin treatment. In all panels, arrows indicate cytoophidium and arrowheads clumps. Scale bars = 10µm.

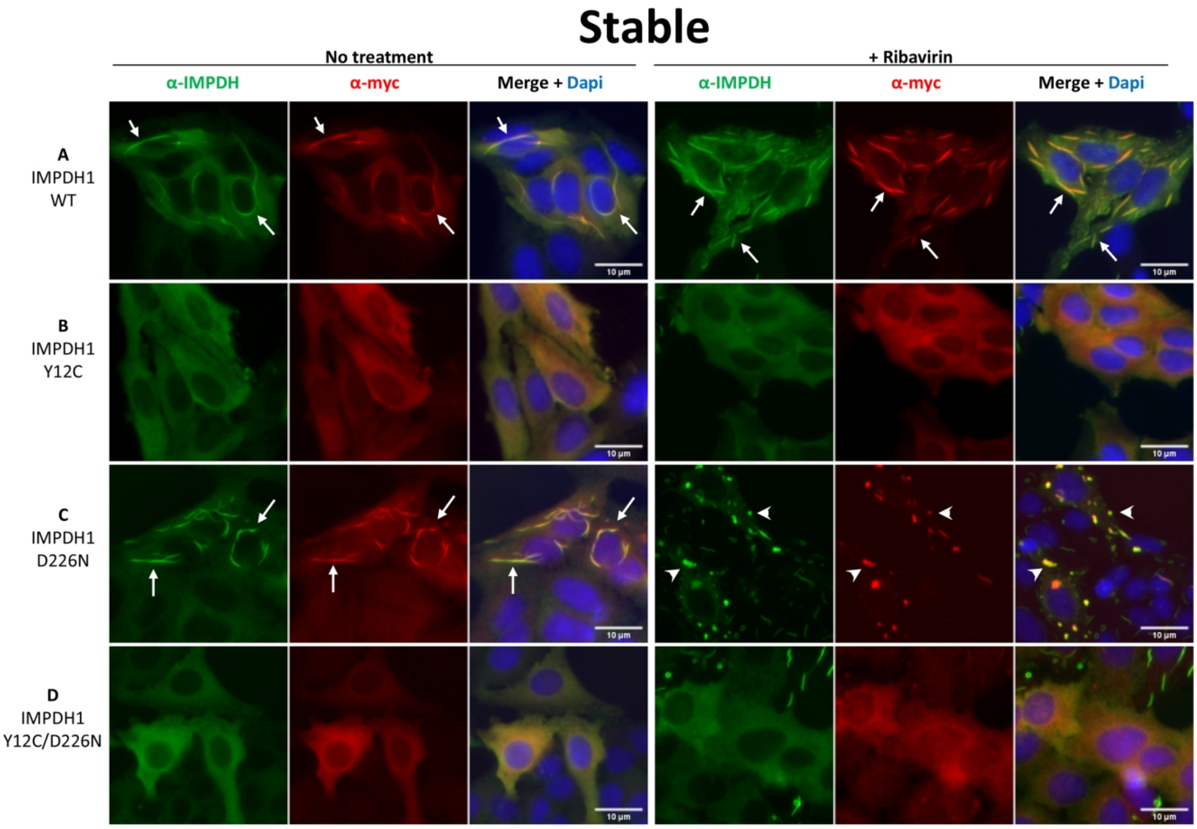

Supp Figure 2. Separate channels for the stable overexpression of IMPDH1 with point mutations. (A-D) The IIF channels of anti-IMPDH antibody labeling (green) plus the anti-myc labeling (red) are shown separately, Merge + DAPI panels are the same as those in Figure 1. Cells were selected with Hygromycin B for 2 weeks before analysis. In all panels, arrows indicate cytoophidium and arrowheads clumps. Scale bars = 10µm.

Supp Figure 3 Shown in the next page →

Supp Figure 3. Analysis of apoptosis in cells with stable expression of IMPDH1-D226N. (A-D) Cells were labeled with Annexin V conjugated to Alexa Fluor 488 and Propidium Iodide for the identification of apoptosis (Quartile 3). (A-C) Annexin V positive controls, HEp-2 cells were exposed to UV for 1 min and analyzed 24h later (B) or treated with staurosporine for 4h before analysis (C). Both treatments increased apoptosis rates ~10x. (D-E) Representative panels for analysis of apoptosis in the cell-lines, with gating for iRFP670 expression. (E-I) Cells were transfected 24h before analysis with a plasmid containing (Flip-GFP), a Caspase3 cleavage sequence (see methods for details). (E) Cells were gated for BFP+ and the proportion of GFP positives (F-G), or the GFP mean fluorescence intensity (MFI) was measured. As positive control, treated with staurosporine for 4h before analysis (G), and the treatment increase ~5x the proportion of GFP+ cells. (J) Quantification of the percentage of apoptosis by Annexin V (Q3 of D) or Flip-GFP, as well as the Flip-GFP MFI, in cells with stable expression of IMPDH1-WT or D226N. The delta (D226N/WT) was calculated in each experimental batch (n=6) and the average is shown (± indicates the Standard Deviation).

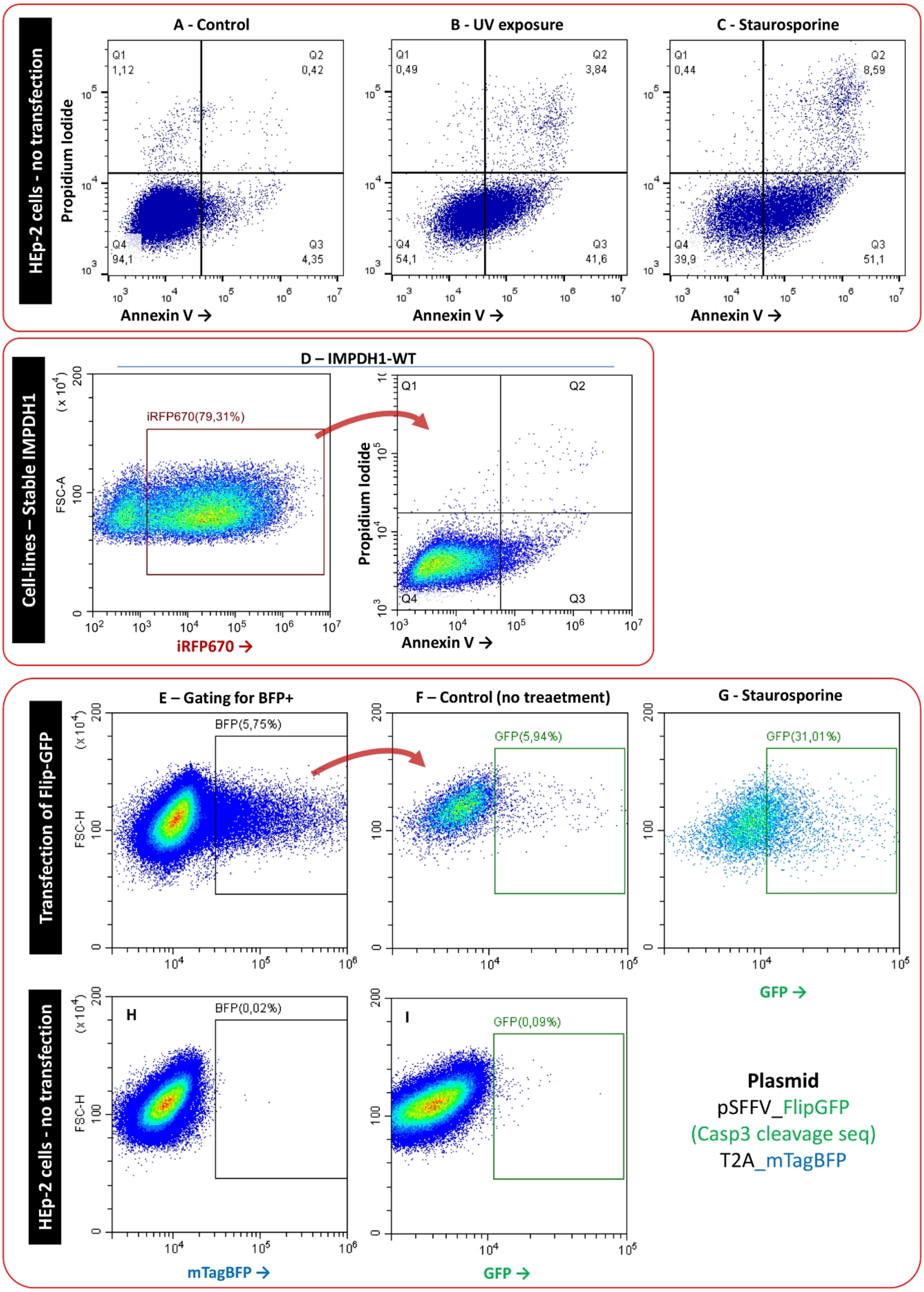

**J - Apoptosis (n=6)**	IMPDH1-WT	IMPDH1-D226N	Delta
% of Annexin V (Q3 in panel D)	4.82% (±0.45)	5.69% (±0.96)	1.27 (±0.30)
% of GFP+ (Flip-GFP)	21.8% (±7.7)	26.6% (±8.8)	1.22 (±0.14)
GFP MFI (Flip-GFP)	16838 (±5332)	17594 (±3817)	1.23 (±0.24)
